# The rs1024611 Regulatory Region Polymorphism Is Associated with *CCL2* Allelic Expression Imbalance

**DOI:** 10.1371/journal.pone.0049498

**Published:** 2012-11-16

**Authors:** Minh-Hieu T. Pham, Gregory B. Bonello, John Castiblanco, Tuan Le, Jose Sigala, Weijing He, Srinivas Mummidi

**Affiliations:** 1 Center for Personalized Medicine, South Texas Veterans Health Care System, San Antonio, Texas, United States of America; 2 Department of Medicine, University of Texas Health, Science Center at San Antonio, San Antonio, Texas, United States of America; University of California San Francisco, United States of America

## Abstract

CC chemokine ligand 2 (CCL2) is the most potent monocyte chemoattractant and inter-individual differences in its expression level have been associated with genetic variants mapping to the cis-regulatory regions of the gene. An A to G polymorphism in the *CCL2* enhancer region at position –2578 (rs1024611; A>G), was found in most studies to be associated with higher serum CCL2 levels and increased susceptibility to a variety of diseases such as HIV-1 associated neurological disorders, tuberculosis, and atherosclerosis. However, the precise mechanism by which rs1024611influences CCL2 expression is not known. To address this knowledge gap, we tested the hypothesis that rs1024611G polymorphism is associated with allelic expression imbalance (AEI) of *CCL2*. We used haplotype analysis and identified a transcribed SNP in the 3′UTR (rs13900; C>T) can serve as a proxy for the rs1024611 and demonstrated that the rs1024611G allele displayed a perfect linkage disequilibrium with rs13900T allele. Allele-specific transcript quantification in lipopolysaccharide treated PBMCs obtained from heterozygous donors showed that rs13900T allele were expressed at higher levels when compared to rs13900C allele in all the donors examined suggesting that *CCL2* is subjected to AEI and that that the allele containing rs1024611G is preferentially transcribed. We also found that AEI of *CCL2* is a stable trait and could be detected in newly synthesized RNA. In contrast to these in vivo findings, in vitro assays with haplotype-specific reporter constructs indicated that the haplotype bearing rs1024611G had a lower or similar transcriptional activity when compared to the haplotype containing rs1024611A. This discordance between the in vivo and in vitro expression studies suggests that the *CCL2* regulatory region polymorphisms may be functioning in a complex and context-dependent manner. In summary, our studies provide strong functional evidence and a rational explanation for the phenotypic effects of the *CCL2* rs1024611G allele.

## Introduction

The CCL2(MCP-1)-CCR2 axis plays a pivotal role in monocyte-macrophage trafficking to sites of inflammation and has been implicated in the pathogenesis of various disease processes such as cardiovascular disease, diabetic nephropathy, rheumatoid arthritis, and several infectious diseases [1], [2], [3], [4], [5], [6], [7], [8], [9]. CCL2 expression levels among individuals are highly variable and this variability may contribute to differential susceptibility to various inflammatory disease states [10], [11]. Such variability in CCL2 expression levels have been ascribed to polymorphisms in the regulatory regions of the *CCL2* gene [10], [11], [12], [13], [14], [15], [16], [17], [18]. In addition, polymorphisms in the *TNF-LTA* locus [19] and a non-synonymous polymorphism in the Duffy Antigen Receptor for Chemokines (DARC) [20] have also been shown to influence serum CCL2 levels.

Rovin et al. initially reported regulatory region polymorphism in the *CCL2* and they found that a polymorphism annotated as rs1024611 (dbSNP database; originally designated as –2518G or –2578G) is associated with increased CCL2 expression [16]. It was subsequently demonstrated that the rs1024611G allele is associated with increased serum CCL2 levels and enhanced leukocyte recruitment to the tissues [10]. Studies emanating from several laboratories confirmed these earlier studies and rs1024611G allele was implicated in increased CCL2 expression levels in serum, plasma, urine and CSF in normal as well as in pathological conditions [11], [12], [13], [21], [22] and in tissues such as liver and skin [14], [18]. However, a number of studies failed to detect rs1024611G allele association with increased serum CCL2 levels [23], [24], [25]. A genome-wide association study (GWAS) that examined protein quantitative trait loci (pQTLs) in serum or plasma from 1200 individuals showed nominal evidence for association of rs1024611 with CCL2 expression levels, although this association did not reach genome-wide significance [26]. It has also been speculated that the increased CCL2 expression from the rs1024611G bearing allele is more pronounced under pro-inflammatory conditions [12].

Cohort-based association studies during the past few years have ascribed a deleterious role to rs1024611G allele with some exceptions. It has been implicated in a myriad of diseases including HIV-1 dementia [10], myocardial infarction and carotid atherosclerosis [11], [27], pulmonary tuberculosis [28] among others (Table 1). However, several other studies have indicated that rs1024611 polymorphism may not play a role in CCL2 expression levels and disease pathogenesis ([25] and Table 1) and it has been argued that most of the earlier studies that looked at the CCL2 expression levels have not taken multiple comparisons into account for their analyses [25]. Thus it is critically important to further dissect the effects of the rs1024611G allele on CCL2 expression and provide a mechanistic basis for its phenotypic effects.

**Table 1:** Disease associations of the *CCL2* rs1024611 polymorphism.

Disease	Association	Reference
Carotid Intima-Media Thickness (IMT)	Yes	[27], [66], [67], [68]
	No	[17], [69]
Atherosclerosis	Yes	[27], [70]
	No	[44]
Myocardial Infarction	Yes	[11]
	No	[71]
Coronary Artery Disease Risk	Yes	[72]
	No	[25]
Pulmonary Tuberculosis	Yes	[28], [54], [55]
	No	[56], [57], [58]
Acute Pancreatitis	Yes	[73]
Systemic Lupus Erythematosus	Yes	[74]
	No	[75], [76], [77]
Lupus Nephritis	Yes	[78], [79]
	No	[80]
Asthma	Yes	[81], [82]
	No	[83]
Hepatic Inflammation and fibrosis	Yes	[18]
Type II diabetes	Yes	[84]
	No	[85]
Metabolic Syndrome	Yes	[86]
Systemic Sclerosis	Yes	[14]
	No	[87], [88]
Crohn’s Disease	Yes	[89]
HIV-1 disease susceptibility	Yes	[10], [90]
HIV-1 Associated Neurological Disorders	Yes	[10]
Alzheimer’s Disease	Yes	[91]
	No	[13], [92], [93]
Breast Cancer	Yes	[94]
Atopic eczema and dermatitis	No	[95]
Hepatitis C infection	Yes	[18]
	No	[96]
Parkinson’s Disease	No	[92]

Previous studies have suggested that the rs1024611 polymorphism mediated its effects via differential binding of various transcription factors and altered transcriptional activity [10], [16], [29], [30], [31]. Reporter assays conducted by various labs to determine differences in the transcriptional strength conferred by this polymorphism yielded variable results. Rovin et al. reported that the –2578G SNP increased transcriptional activity of the *CCL2* distal enhancer [16]. By contrast, other studies suggested a reduced transcriptional activity associated with this SNP [23], [30]. One caveat of the aforementioned experiments is that these studies fail to take into consideration the potential role of the linked polymorphisms on transcription factor binding as well as transcriptional activity. Thus the functional basis of interindividual variation in CCL2 expression is complex and remains unresolved.

A powerful way to detect allelic differences in expression is to quantify the expression of transcripts derived from each individual allele in a heterozygous state [32], [33], [34]. This approach is based on the premise that in the absence of genetic or epigenetic variation there will be equal amount of expression from both the maternal and paternal alleles as they are exposed to identical intracellular environment. By contrast, individuals heterozygous for *cis*-acting polymorphisms that affect gene expression or mRNA processing or differences in their epigenetic signatures will show an altered level of mRNA expression originating from one allele compared with its partner allele. This phenomenon referred to as allelic expression imbalance (AEI) can serve as an integrative quantitative measure of effects of *cis*-acting and epigenetic variation [34]. We therefore hypothesized that the rs1024611G allele which is associated with strong phenotypic effects may exhibit increased expression in a heterozygous state. For this purpose, we performed extensive haplotype analysis of the *CCL2* locus that identified a polymorphism in its transcribed region that showed complete linkage disequilibrium with rs1024611 that we successfully employed to elucidate AEI of *CCL2.* Our results conclusively demonstrate that haplotype containing rs1024611G allele is associated with increased expression in a heterozygous state providing a strong validation for its biological effects seen in cohort based studies.

## Results

### A Transcribed SNP can Serve as a Proxy for rs1024611

**Figure 1:**
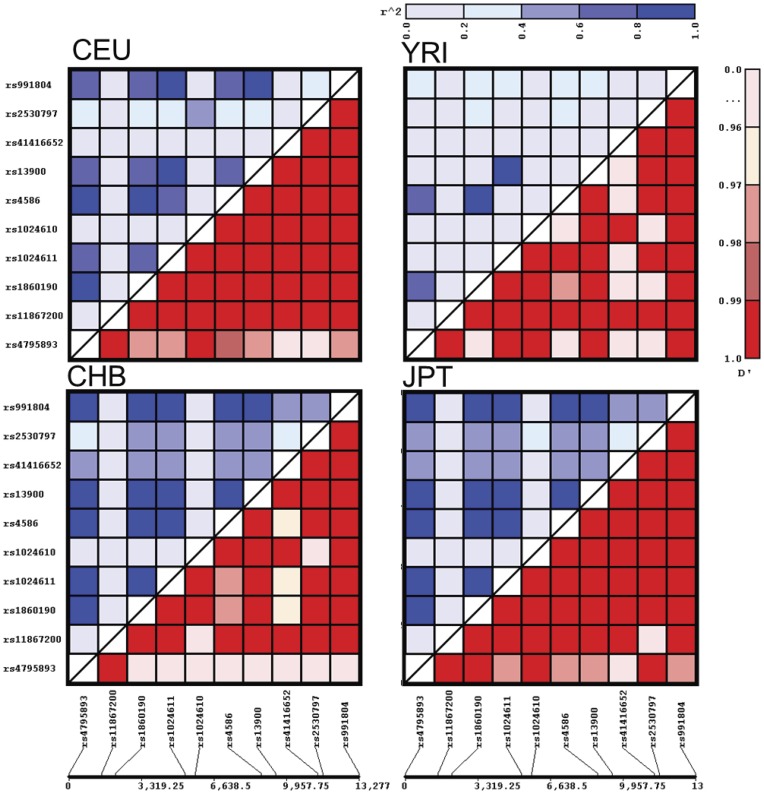
Linkage disequilibrium plots for the shared polymorphisms in the *CCL2* locus. In all, 9 different polymorphisms that are located within a 25 kb region that spans the rs1024611 polymorphism are shown. The heatmaps display pairwise r^2^ (above diagonal) or D’ (below diagonal) for the each pair of polymorphisms. The relative physical distance between the markers is shown in the bottom of the plots. Populations shown are Yoruba in Ibadan, Nigeria (YRI), Japanese in Tokyo, Japan (JPT), Han Chinese in Beijing, China (CHB) and CEPH (Utah residents with ancestry from northern and western Europe, CEU) populations (HapMap data release #28 August 2010 on Build 36). Only unrelated individuals were used for the analysis. LD maps were constructed using the JLIN program [61].

The rs1024611 polymorphism is located in the *CCL2* enhancer region and thus cannot be used to assess differential expression of the transcribed alleles. To identify any possible SNPs in the transcribed region that can serve as a proxy for this polymorphism, we generated linkage disequilibrium (LD) maps of the *CCL2* locus. For this, we used publicly available SNP information from a region that spanned a 25 kb region across the rs1024611 polymorphism (www.hapmap.org) [35]. Genotype data from Utah residents with Northern and Western European ancestry from the CEPH collection (CEU), Han Chinese from Beijing, China (CHB), Japanese from Tokyo (JPT), and Yoruban from Ibadan, Nigeria (YRI) was used for creating the LD map that included *CCL2* far upstream region, enhancer, promoter, open reading frame, introns, 3′UTR and 3′-flanking region. Data was analyzed and visualized by using JLIN program and only polymorphisms that are shared by all the populations examined were included in the analysis (Fig. 1). The upper half of each LD plot for each denoted population that is depicted in various shades of blue represents the r^2^ (R-square) which is square of the correlation coefficient of a given marker pair. An r^2^ equal to 1 indicates complete pairwise LD between the markers in question. The lower-half of each LD plot represents the D′ (D-prime) which indicates normalized covariance for a given marker pair and values close to 1 indicate strong LD. Chromosomal position and location of the genetic variants used to generate the LD profiles for the Hapmap populations that are depicted in Fig. 1 are shown in Table 2. All the SNPs examined did not deviate from the expected Hardy-Weinberg proportions in each population that was analyzed (Table 3). The highest frequency of the rs1024611G allele was observed among Asian ancestry populations (CHB and JPT) followed by European ancestry CEU population. The African ancestry population showed the lowest frequency. We observed a strong high LD between rs1024611 and several other polymorphisms in *CCL2* with r^2^ close to 1 (Table 4). The highest r^2^ values were observed for rs13900 and rs991804 (Table 4). Of note, the SNP at rs13900 maps to the transcribed region of the *CCL2*. Both the pairwise r^2^ and D′ values for rs1024611, rs13900 and rs991804 was equal to 1, suggesting that they can act as proxies for each other.

**Table 2:** Chromosomal positions and genomic location of the *CCL2* locus HapMap SNPs.

SNP ID	Chr. Position(hg18)	Location	Variation	AncestralAllele
rs4795893	29598561	Inter-genic	G/A	A
rs11867200	29600082	Enhancer	C/T	C
rs1860190	29600686	Enhancer	T/A	T
rs1024611	29603901	Enhancer	A/G	A
rs1024610	29604344	Enhancer	A/T	T
rs4586	29607382	cds-synonymous	C/T	C
rs13900	29608024	3′UTR	C/T	C
rs41416652	29609974	Inter-genic	T/C	T
rs2530797	29610207	Inter-genic	A/G	A
rs991804	29611838	Inter-genic	A/G	G

CCL2, CC Chemokine ligand 2; SNP, Single nucleotide polymorphism; cds-coding sequence; hg18 corresponds to NCBI build 36.1 (Release Date Mar. 2006). Ancestral allele shown is from the dBSNP database (www.ncbi.nlm.nih.gov/projects/SNP/ *).*

**Table 3:** Relative minor allele frequencies of *CCL2* HapMap SNPs in different populations.

SNP ID	MAF(HWEp)			
	CEU(n = 112)	CHB(n = 84)	JPT(n = 86)	YRI(n = 116)
rs4795893	0.6429(0.41)	0.3735(0.82)	0.3779(0.65)	0.3147(0.195)
rs11867200	0.2232(0.79)	0.0952(0.15)	0.0756(1.0)	0.0086(0.99)
rs1860190	0.6518(0.40)	0.3750(1.0)	0.3663(1.0)	0.2414(0.46)
rs1024611	0.2991(0.04)	0.6250(1.0)	0.6279(0.49)	0.1983(0.39)
rs1024610	0.1830(0.99)	0.0536(1.0)	0.0814(1.0)	0.0517(1.0)
rs4586	0.6473(0.54)	0.3750(1.0)	0.3706(0.49)	0.2414(0.46)
rs13900	0.2991(0.04)	0.2991(1.0)	0.6279(0.49)	0.2026(0.56)
rs41416652	0.9777(0.99)	0.5060(0.99)	0.5174(0.28)	0.9911(0.99)
rs2530797	0.6518(0.84)	0.8012(0.99)	0.7674(0.03)	0.8922(0.36)
rs991804	0.2991(0.04)	0.6310(0.99)	0.6279(0.49)	0.4267(0.13)

The minor allele frequency as well as the Hardy-Weinberg equilibrium p-values (in parenthesis) for the different SNPs in each population are indicated.

CCL2, CC chemokine ligand 2; SNP, Single nucleotide polymorphism; CEU, Utah residents with Northern and Western European ancestry from the CEPH collection (CEPH, Centre du Etude Polymorphisme Humain); CHB, Han Chinese from Beijing, China; JPT, Japanese from Tokyo, Japan; YRI, Yoruban from Ibadan, Nigeria; the numbers in parenthesis next to the each population group show the genotyped individuals included in the analysis.

**Table 4:** Linkage disequilibrium between rs1024611 and other SNPs in the *CCL2* locus.

SNP ID	Distance (bp)	r^2^
rs4795893	−5340	0.73
rs11867200	−3819	0.124
rs1860190	−3215	0.792
rs1024610	+443	0.093
rs4586	+3481	0.778
**rs13900**	**+4123**	**1.0**
rs41416652	+6173	0.0535
rs2530797	+6306	0.231
**rs991804**	**+7937**	**1.0**

Single nucleotide polymorphism, SNP; bp, basepairs; CCL2, CC chemokine ligand 2; r^2^, square of the correlation coefficient between rs1024611 and the indicated SNP.

Haplotype analysis using these ten polymorphisms revealed haplotypes that are shown in Table 5. A total of 22 haplotypes were predicted in all the populations of which only 10 had a greater than 2% frequency in any given population. Haplotype 3 (h3), h4, and h11 contained the rs1024611G allele (Table 5). The frequency of the haplotype(s) bearing the rs1024611G allele in the East Asian population was greater than 60% whereas in CEU the frequency was 28%. Interestingly, h4 was more frequent in CHB and JPT whereas h3 was more common in CEU and YRI populations. h3, h4, h13, and h18 contained the rs13900, but as noted the frequency of both h13 and h18 is<1% (Table 5). Thus rs13900 is mainly restricted to the rs1024611G-bearing h3 and h4. In addition, both h3 and h4 contain the rs1860190T allele which is in the far upstream region which they share with both h16 and h17 that are restricted to the YRI population. Our results are in general agreement with previously reported haplotypes of the *CCL2* locus [36] and their frequency [11].

**Table 5:** Inferred haplotypes in the *CCL2* locus and their frequency.

Haplotype	SNP ID										Hapmap population and haplotype frequencies			
	rs4795893	rs11867200	rs1860190	rs1024611	rs1024610	rs4586	rs13900	rs41416652	rs2530797	rs991804	CEU(n = 224)	CHB(n = 164)	JPT(n = 170)	YRI(n = 222)
h1	G	T	A	A	A	T	C	C	G	G	0.22 (50)	0.01(16)	0.07(12)	0.01(2)
h2	G	C	A	A	T	T	C	C	A	G	0.18(41)	0.04(7)	0.08(14)	0.02(4)
h3	A	C	T	G	A	C	T	C	G	A	0.28(62)	0.13(22)	0.15(25)	0.19(43)
h4	A	C	T	G	A	C	T	T	G	A	0.02(4)	0.48(78)	0.47(80)	0 (0)
h5	A	C	T	A	A	C	C	C	G	G	0.05(11)	0 (0)	0 (0)	0.25(56)
h6	G	C	A	A	A	C	C	C	A	G	0.16(36)	0.15(24)	0.15(25)	0.08(17)
h9	G	C	A	A	A	T	C	C	G	G	0.07(16)	0.07(12)	0.06(11)	0.09(20)
h15	G	C	A	A	A	T	C	C	G	G	0 (0)	0 (0)	0 (0)	0.03(7)
h16	G	C	T	A	A	C	C	C	G	G	0 (0)	0 (0)	0 (0)	0.08(17)
h17	A	C	T	A	A	C	C	C	G	A	0 (0)	0 (0)	0 (0)	0.22(49)
Anc.	A	C	T	A	T	C	C	T	A	G				

Only haplotypes with frequency greater than 2% in any population are shown. Data were downloaded from the Hapmap web site (http://hapmap.ncbi.nlm.nih.gov/) and haplotypes inferred using the ARLEQUIN program. Only the reference polymorphisms that were shared by all the population groups examined were used to generate the haplotypes. Population description is as in Table 3. The numbers within the parenthesis indicate the number of individuals (n). The h18, h19, h20, h21, h22 were present exclusively in the YRI each with a frequency of 0.0045. h7 and h8 were present exclusively in CEU each with a frequency of 0.0045. h12 and h13 were present exclusively in CHB each with a frequency of 0.0061. h14 was exclusively present in JPT at a frequency of 0.0059. The haplotype frequency of h10 was 0.005 in CEU, 0.006 in CHB, and 0.009 in YRI. The haplotype frequency of h11 was 0.005 in CEU, 0.012 in CHB, and 0.012 in JPT. The ancestral state of the reference SNPs (Anc.) was obtained from the dBSNP database (http://www.ncbi.nlm.nih.gov/projects/SNP/).

### Validation of Pyrosequencing Technique to Verify AEI of CCL2

**Figure 2:**
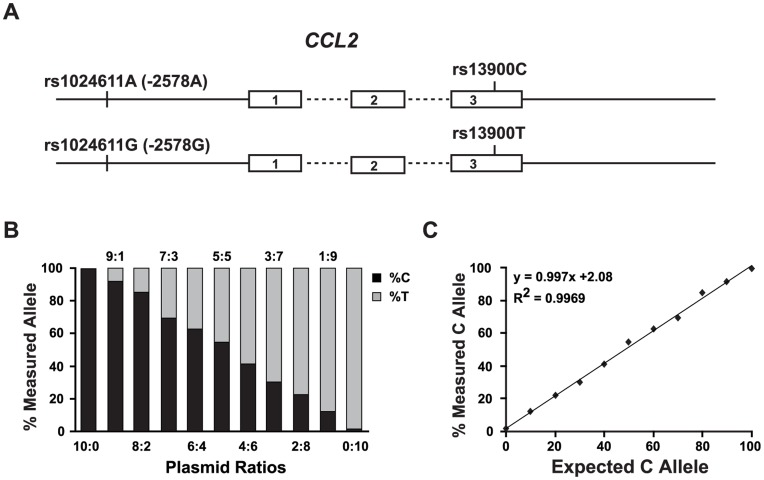
Validation of a pyrosequencing assay to measure AEI at rs13900 polymorphism. A. Schematic of the *CCL2* gene structure and the LD between regulatory region polymorphism rs1024611 and the transcribed polymorphism rs13900. Numbered boxes are exons and the dashed lines connecting the exons are the introns. rs1024611 is located 2578 bp upstream of the *CCL2* translational start site and rs13900 is located in the 3′UTR. B. Stacked bar graph represents the levels of the C (black) and T (grey) alleles in PCR products as determined by pyrosequencing. The PCRs were performed on the plasmid mixtures containing rs13900C and rs 13900T allele combined at the indicated ratios to simulate homozygous (10∶0 and 0∶10) and heterozygous samples (5∶5) as well as different allelic levels (other indicated ratios). Data shown is from one representative experiment from three independent experiments which gave similar results. C. Regression analysis of amplification products obtained from the rs13900 C- and rs13900 T-bearing plasmids combined at different proportions. The measured levels of the C allele (*y-axis*) were plotted against the expected levels (*x-axis*). There was a near linear relationship between these values (R^2^ = 0.9969) suggesting that the pyrosequencing can serve as a sensitive assay to measure the levels of rs13900C and rs13900T alleles in heterozygous individuals. Similar results were obtained with using genomic DNA mixtures from homozygous and heterozygous individuals (data not shown).

Our LD analysis of *CCL2* locus suggested that rs13900 C/T alleles can serve as a proxy for the rs1024611 A/G, respectively and could be targeted to assess allele-specific differences in expression (Fig. 2A). Pyrosequencing has been used extensively used to assess AEI for several genes [37] and we adapted this technique to assess the relative contribution of each allele to CCL2 transcript levels in heterozygous donors. The expression level of a specific allele as detected during pyrosequencing is reflected as the height of peak corresponding to that specific nucleotide in the pyrogram. We validated the pyrosequencing technique for the rs13900 to verify that the peaks in the pyrogram reflect the actual proportions of the C and T variants. For this we cloned *CCL2* 3′UTR from the donors who were homozygous for rs13900 C or T variants. We mixed the plasmids at specific concentrations and subjected the mixtures to PCR amplification and pyrosequencing (Fig. 2B). No or minimal amplification was detected when only one allele was used for amplification. The peaks of the pyrograms for the %C and %T obtained from the mixtures was in accord with the ratio of the plasmids in the template. To determine if there is a correlation over the range of concentrations tested, we plotted the expected and measured peak percentages and used linear regression analysis. The r^2^ was>0.99 suggesting that the pyrosequencing assay that we developed is highly quantitative and could be used for accurate assessment of the allelic differences in gene expression (Fig. 2C).

### Assessment of AEI in the CCL2 in Human Primary Cells, Brain, and Transformed Cell Lines

**Figure 3:**
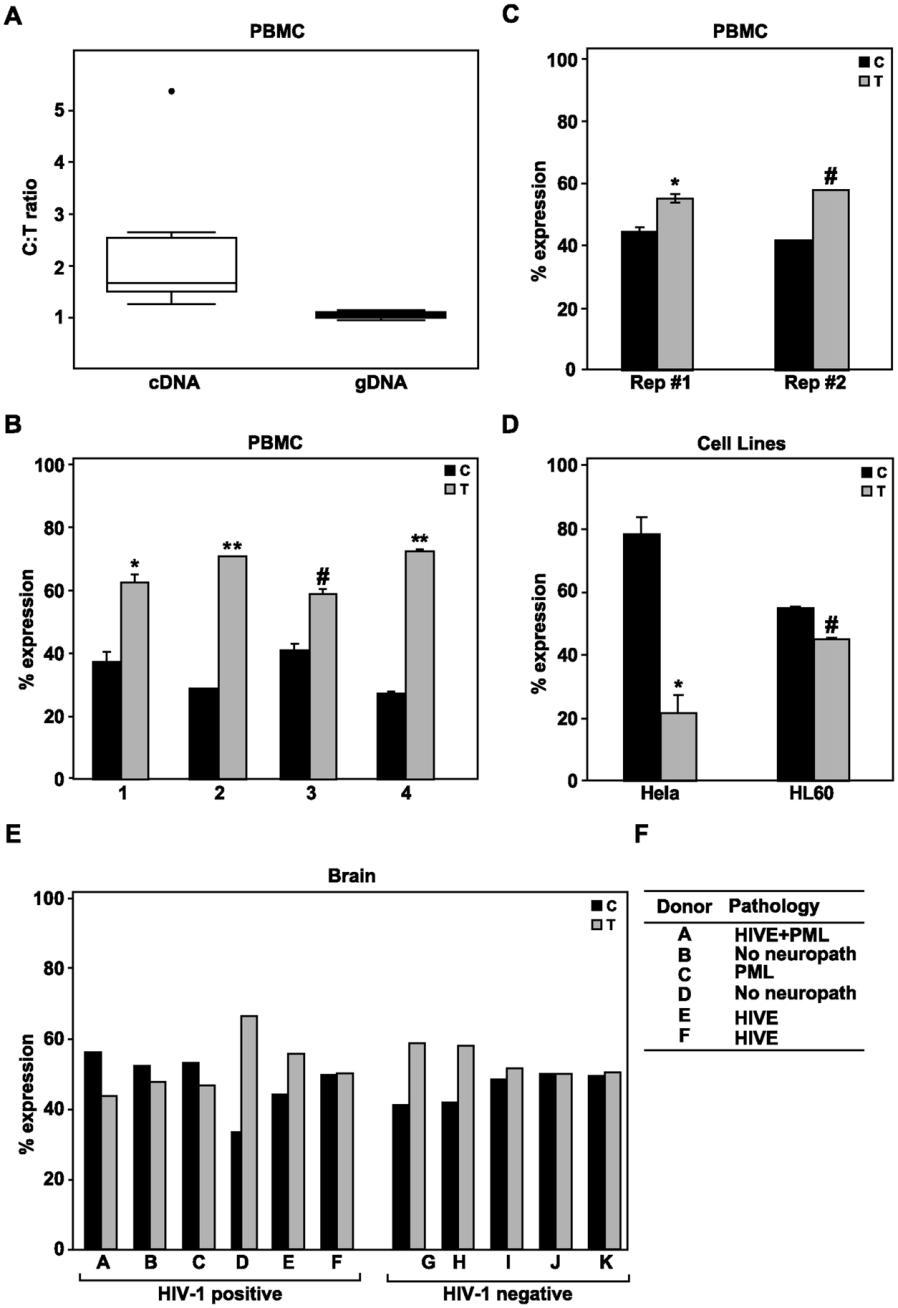
AEI of CCL2 in PBMC, cell lines and brain. A. Allelic ratios for cDNA and gDNA were determined by pyrosequencing in eight independent donors who were heterozygous for rs13900 and rs1024611 polymorphisms. RNA was extracted from PBMCs treated with LPS for 3 h. and cDNA was synthesized. Pyrosequencing was performed as described under Methods. The ratios of expression (C *vs.* T) are log_2_-transformed and are shown on the y-axis. Statistical significance was determined using a two-tailed Wilcoxon rank sum test (p = 0.0009). B. Stability of allele-specific differences in the CCL2 expression in LPS treated PBMC from heterozygous individuals. AEI was assessed at three different times in 4 independent donors over a period of 4–6 months with a gap of at least 2 weeks between experiments in a single donor. The y-axis indicates the percent level of expression of the C or the T allele. C. AEI in nascent RNA. LPS treated PBMC were cultured in presence of ethylene uridine (EU). The EU RNA was subjected to a click reaction that adds a biotin handle which is then captured by streptavidin beads. cDNA was synthesized from the captured nascent RNA and PCR amplified and subjected to pyrosequencing. Data shown are from two independent biological replicates from a single donor. D. rs13900C allele is expressed at higher levels in heterozygous cell lines. Differential expression of CCL2 alleles in heterozygous cell lines (HeLa- cervical cancer cell line; HL60-myeloid leukemia cell line). cDNA was synthesized using RNA extracted from LPS treated cell lines and PCR and pyrosequencing were performed. E. AEI in brain tissue. RNA was extracted from post-mortem brain tissues obtained from HIV-1 infected and normal donors and the extent of AEI in CCL2 was assessed in heterozygous donors by pyrosequencing. F. Clinical features and pathology associated with the HIV-1 positive donors. HIVE- HIV encephalitis; PML-Progressive Multifocal Leukoencephalopathy; Donors B & D did not exhibit any neuropathology. Statistical significance for differences in the levels of expression between the alleles was calculated using a paired t-test (*, p<0.05, **, p<0.001, #, p<0.0001).

Having established that we can accurately quantify allele-specific expression in *CCL2* gene we examined the degree of AEI in individuals who were heterozygous for the rs1024611 polymorphism. Total RNA and genomic DNA were isolated from PBMC from eight healthy human volunteer donors after they were treated with LPS for 3 h to induce CCL2 expression. The induction of CCL2 following lipopolysaccharide treatment was confirmed by qRT-PCR (Fig. S1). We then quantified the levels of the rs13900 C or T alleles in the transcript by subjecting the RT-PCR products to pyrosequencing. As a control, genomic DNA was also amplified from these donors and was subjected to pyrosequencing (Fig. 3A). All the donors who were heterozygous for rs1024611 were also heterozygous for rs13900. Boxplots illustrate striking differences in the detected levels of the C and T alleles in cDNA and gDNA suggesting that the *CCL2* is subjected to AEI and that the expression level of the rs13900T allele (that is present in all the haplotypic phases containing rs1024611G) is relatively higher when compared to the allele bearing rs13900C allele (that is present in most haplotypic phases containing rs1024611A), respectively. We further confirmed that the allelic differences in CCL2 expression detected in LPS treated PBMC were also reproducible in similarly treated purified monocytes (Fig. S2).

We then asked if these differences in allele expression are stable within an individual over a period of time. To assess this we used three independent samples of RNA from four different donors and determined the relative expression of the rs13900C or rs13900T alleles. Our data suggested that all four donors consistently maintained higher expression of rs13900T allele relative to the C allele suggesting that AEI of *CCL2* is highly stable (Fig. 3B). We reasoned that the differences in the expression levels between the two alleles in the heterozygous donors may be due to either transcriptional or post-transcriptional mechanisms. Therefore we tested AEI in newly transcribed RNA that was isolated from PBMC following LPS treatment. Nascent RNA was captured as described under Methods and pyrosequencing was performed to detect the relative levels of rs13900C or rs13900T alleles (Fig. 3C). We found that differences in the expression level of the rs13900T-bearing allele versus the rs13900C-bearing allele is similar to the levels seen in total RNA suggesting that the imbalance in the *CCL2* allele expression most likely occurs at the transcriptional level although post-transcriptional mechanisms that alter transcript stability cannot be completely ruled out.

Genome-wide studies have provided rich data for linking polymorphisms with gene expression. We queried the Sanger gene expression database to assess the role of heterozygosity at rs1024611 polymorphism (rs13900 not used as it is not included in the database) in CEU (B-cells), T cells, fibroblasts, and fat tissue [38], [39]. Our analysis of these data indicated that there were no significant differences between the RNA expression levels of homozygous individuals carrying rs1024611A SNP and heterozygous individuals. We also examined the AEI in two heterozygous cell lines HeLa and HL60 and surprisingly we detected increased expression of the C allele relative to T allele (Fig. 3D). Taken together, our results suggest that AEI of *CCL2* may be tissue-specific and cell-type specific and as noted before cannot be extrapolated between different studies and therefore need to be cautiously interpreted in the context of the disease pathogenesis.

CCL2 plays a key role in the recruitment of cells of monocyte/macrophage lineage to the brain and our group has previously shown that the presence of rs1024611G allele (–2578G) that leads to increased recruitment of mononuclear phagocytes in tissues is associated with increased risk to HIV-1 associated dementia [10]. Ragin et al. reported that CCL2 is the most prominent correlate of neurological injury in HIV-1 infected patients [40]. This prompted us to assess the extent of AEI in brain tissue obtained from HIV-1 positive and negative donors (Fig. 3E). We found that there was increased expression of rs13900 T in the post-mortem brain tissue obtained two HIV-1 positive donors (donors D & E) and two normal donors (G & H). In other samples the expression level of C allele was slightly higher or both alleles were present at similar levels. This lack of consistent AEI in brain from heterozygous individuals may be attributed to the cellular composition of sampled brain regions or other variables. Nevertheless, these findings suggest that *CCL2* gene expression in brain may also be subjected to AEI and potentially could explain the increased CCL2 levels seen in CSF in individuals bearing rs1024611G allele and their increased susceptibility to HIV-1 related neurological disorders.

### Mechanistic Basis of AEI in CCL2 Locus

As previously discussed, most of the studies that examined the functional effects of rs1024611 allele have yielded conflicting results. This raised the possibility that other SNPs that are in LD with rs1024611G allele may modulate its expression. Our previous studies on *CCL2* gene regulation support such a notion as there is a high degree of sequence as well as functional conservation in the *CCL2* far upstream region. Haplotype analysis showed a high r^2^ between rs1860190 and rs1024611G alleles. rs1860190 is located ∼3.2 kb upstream of rs1024611 and potentially could influence transcriptional activity (Table 4). Two additional polymorphisms that are designated as rs2857654 and rs2857656 are located ∼250 bp upstream and ∼2.2 kb downstream of the rs1024611, respectively, could also be involved in AEI as they have r^2^>0.9 in CEU population. Please note that the latter two SNPs were not included in the haplotype maps shown in Fig. 1 and Table 5 due to their restricted population distribution.

**Figure 4:**
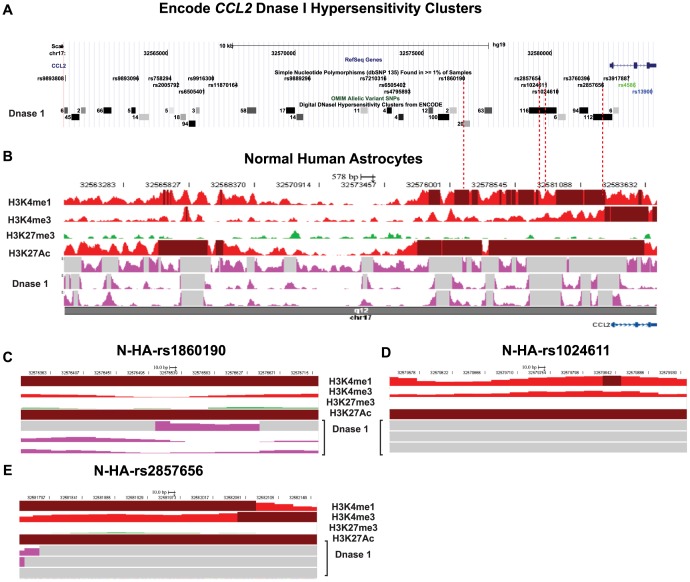
Epigenetic features associated with *CCL2* locus in cell lines and normal human astrocytes. (A–B) Relative location of the SNPs linked to rs13900 (highlighted by red dashed lines) to Encode DNase 1 hypersensitivity sites in the *CCL2* locus as depicted in the UCSC genome browser (Panel A) and histone and DNase 1 tracks in the human epigenome browser (Panel B). Nucleotide numbering is according to hg19. The DNase 1 sites in panel A are depicted as boxes. The shade of the box is proportional to the signal strength detected with darker shaded boxes representing increased sensitivity to digestion. The numbers next to the boxes indicate the numbers of cell lines in which the region is hypersensitive. Panel B shows a wiggle plot depicting relative enrichment of the histone activation markers (H3K4me1, H3K4me3, H3K27Ac, indicated in red) and histone repressive marks (H3K27me3, indicated in green) across the *CCL2* locus. The heatmap track was configured to set the threshold for the peaks at 20 and values higher than the threshold are shown in brown. Also shown are the tracks for DNAse 1 sensitivity (purple tracks). Regions in gray indicate the regions with higher peaks than the set threshold. (C–E) Heatmaps showing localized histone tracks in *CCL2* 5′-flanking regions that overlap with the linked polymorphisms. A 500 bp region that spans the indicated polymorphism is shown. No separate panel is shown for rs2857654 due to its proximity to rs1024611. Other details are as in Panel B. The source of the data used for the generation of the DNase 1 tracks is from the Geo accession number GSE29692 (DNaseI Hypersensitivity by Digital DNaseI from ENCODE/University of Washington; public release on June 03, 2011) and for the histone tracks is Geo Accession numbers GSM733763 (H3K27Ac), GSM733729 (H3K27Me), GSM733747 (H3K4me3), and GSM733710 (H3K4me1) deposited by the Bernstein Lab at the Broad Institute (Histone Modifications by ChIP-seq from ENCODE/Broad Institute; public release on Jun 2, 2011). The tracks were generated using ENCODE database and UCSC genome browser [63], [64] and Human epigenome browser [65].

We used two complementary approaches to determine the functional relevance of the regions that contain the SNPs that are linked to rs1024611. In the first approach, we took advantage of the publicly available whole genome ChIP-Seq and DNaseI hypersensitivity data to examine epigenetic profiles associated with these regions which might provide clues for their functional relevance. *Cis*-regulatory regions that either have a promoter-like or enhancer-like activity are often characterized by enrichment or depletion of specific post-translational histone modifications and increased DNase I hypersensitivity. For example, the enrichment of histone modification H3K4me3 marks the promoters whereas enrichment of the histone H3K4me1 and depletion of H3K4me3 marks the enhancers. Our analysis revealed the presence of multiple DNAse1 hypersensitivity regions in numerous cell lines as well as specific histone signature patterns in the *CCL2* locus (Fig. 4). The polymorphisms rs2857654, rs1024611, and rs2857656 colocalize to regions that exhibit DNase 1 hypersensitivity in>100 cell types (Fig. 4A). The rs1860190 is located in a region that showed moderate level of hypersensitivity in 20 different cell types (Fig. 4A). There was strong enrichment of the H3K4me1 in the regions bearing rs1860190, rs1024611/rs2857654, and rs2857656 in normal human astrocytes (Fig. 4B). In addition, these regions also show strong enrichment of H3K27Ac that is thought to mark active enhancers and distinguish them from poised enhancers (Fig. 4B) [41]. As expected, there was a marked depletion of the H3K27me3 modification which is normally associated with repressive chromatin (Fig. 4). We also found that with a few exceptions the DNAse 1 hypersensitivity in the *CCL2* correlates with the levels of H3K4me1 and H3K27Ac in normal human astrocytes (Fig. 4). These findings reaffirmed that several of the SNPs linked to rs1024611 are present in *CCL2* regulatory regions that contain epigenetic marks associated with active transcription and could potentially modulate its regulation.

**Figure 5:**
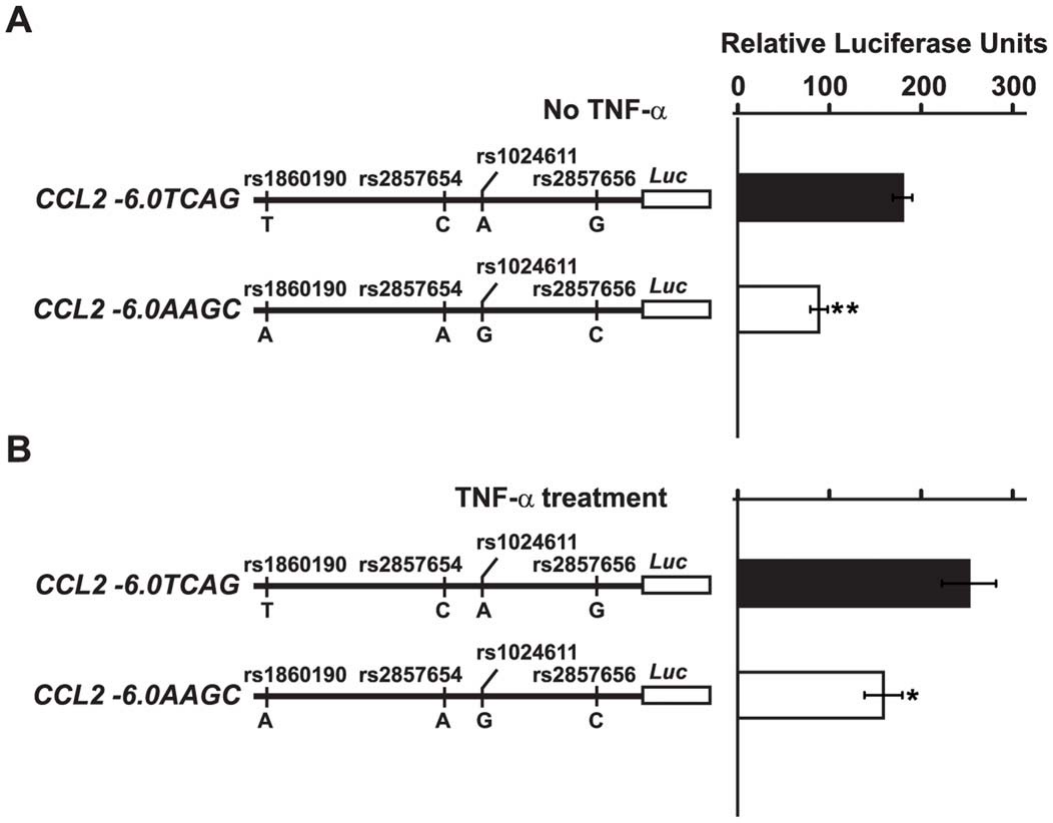
Transcriptional effects of the *cis*-regulatory region SNPs that are in LD with rs1024611. (A–B). On the left is the schematic of the *CCL2* haplotype-specific constructs that were examined for differences in transcriptional strength. The following pair of constructs bearing the indicated polymorphisms, *CCL2 -6.0TCAG* and *CCL2 -6.0AAGC* (rs1860190T, rs2857654C, rs1024611A, rs2857656G and rs1860190A, rs2857654A, rs1024611G, rs2857656C, respectively), were tested. The constructs were obtained from a single heterozygous donor exhibiting AEI. The constructs were transfected into U87MG astroglioma cells and were tested at basal level (panel A) and following TNF-α treatment (panel B). Luciferase activity was determined as described in the *Methods*. The relative luciferase units refer to the fold increase in activity obtained from the *CCL2 -6.0* constructs relative to that obtained with the promoterless pGL4.16 vector. The data shown were obtained from 7 independent experiments and the error bars indicate the standard error of mean and statistical significance was calculated using two-tailed Student’s *t* test (**, p<0.0001, *, p = 0.02).

To directly test this hypothesis, we generated haplotype-specific constructs to determine the role of the linked polymorphisms on *CCL2* transcriptional strength. Of note, these constructs were generated from a heterozygous donor who exhibited AEI. The reporter constructs spanned ∼ 6.0 kb upstream of the *CCL2* transcriptional start site which included the SNPs rs1860190, rs2857654, rs1024611, rs2857656 under basal as well as under stimulatory conditions (Fig. 5). Haplotype-specific reporter constructs were transfected into U87MG which is an astroglioma cell line that expresses CCL2 and has been extensively used previously to study its regulation [42], [43]. Unexpectedly, we found that the reporter vector bearing the rs1024611G allele had a lower transcriptional activity when compared to the reporter vector containing the rs1024611A allele (Fig. 5). We also tested these constructs in normal human astrocytes and found that there was no significant difference in transcriptional strength between these constructs (Fig. S3). Our results suggested that there is discordance between the in vitro reporter assays and in vivo allelic differences in expression.

## Discussion

Studies in knockout mice as well as overexpression studies have established the preeminent role of CCL2 as a monocyte chemoattractant as well as its non-redundant role during the inflammatory response [4]. The discovery of polymorphisms in *CCL2* cis-regulatory region has provided a strong impetus to examine their role in association studies covering a wide spectrum of inflammatory diseases. Remarkably, in contrast to several widely studied polymorphisms, there is strong in vitro and in vivo experimental evidence linking the rs1024611G allele to CCL2 expression levels as well as leukocyte recruitment to the inflammatory sites [10], [14]. However, till date, no unifying molecular mechanism has been proposed that convincingly explains the phenotypic effects of this polymorphism. In this study, we provide a molecular explanation for the phenotypic effects and disease associations of rs1024611G allele and the significance of our findings is discussed further below.

### Linkage Disequilibrium in the CCL2 Locus

We have developed an extensive linkage map spanning ∼25 kb region of the *CCL2* locus including far upstream region, distal enhancer, and promoter as well as the transcribed region. Most studies till date have focused on a limited span of *CCL2* locus for generating the haplotype maps [11], [25], [44]. The extended haplotype maps have allowed us to confirm that the rs1024611G SNP is in complete linkage disequilibrium with the transcribed rs13900 and help identify other potential SNPs in the locus that may be of functional relevance. Our results also agree with other reported haplotype maps that indicated strong LD between rs1024611 and rs13900 [36]. Of note, there was a high degree of linkage between rs1024611G and rs13900T alleles in all the populations examined, suggesting that rs13900C>T can be used to assess AEI in various population groups.

### AEI of CCL2

This study provides a striking example of AEI as a powerful means to capture biologically meaningful variability and a functional assay to resolve conflicting phenotypic data. Our results from LPS stimulated PBMC from eight independent donors and repeated measurements from four donors consistently showed a higher expression level of transcript containing rs13900T. These studies also provide a cautionary note about inferring allele-specific differences using expression data from transformed cell lines as we could not detect the same in both HeLa and HL60 cell lines which also show robust CCL2 expression. Several of the genome-wide studies that used transformed lymphoblastoid cells (which have a very low expression levels of CCL2) and primary cells and tissues such as skin, fat, fibroblasts, and T cells also failed to detect significant differences between homozygous rs1024611A and heterozygous donors with respect to CCL2 expression levels [38], [39]. This can be attributed to different tissues sampled and/or the technology platforms used. Johnson et al. examined imbalance of CCL2 expression in heart and monocytes using rs4586 polymorphism in the coding region and detected AEI ratios of 0.67–1.87 [45]. They concluded that AEI observed with rs4586 is incompatible with the heterozygosity at rs1024611. This is not a surprising result because the r^2^ between rs4586 and rs1024611 is 0.778. While it could be argued that PBMC is a mixed population and cells belonging to multiple lineages could be contributing to CCL2 expression levels and thus to the AEI, monocytes are known to be the most predominant source of CCL2 from PBMC [1], [46]. In support of this, in qRT-PCR experiments to measure CCL2 in sorted cell populations, we found that the Ct values obtained from activated T-cells were>30, whereas the Ct values obtained with LPS treated monocytes were<22. As AEI detection needs robust expression levels [45], we did not further pursue it in fractionated cell populations.

### Proinflammatory Conditions and their Contribution to CCL2 AEI

We have used mRNA isolated from LPS treated PBMC for assessing AEI as it is a potent inducer of CCL2 expression in cells of monocytic lineage. We have not evaluated other inducers of CCL2 expression such as IFN-γ and IL-1β in this study. However, studies from other laboratories have shown that increased CCL2 expression is associated with rs1024611G allele under a variety of stimulatory conditions such as IL-1β [12], [16], LPS [12], *Mycobacterium tuberculosis* antigens [28], and TNF-α [14]. Thus it is plausible that a common signal transduction pathway shared by several of the inflammatory cytokines/stimuli is responsible for differential expression of the allele linked to rs1024611G allele. On the other hand, we cannot completely rule out allele-specific differences in CCL2 expression at basal level in absence of stimulation. However, testing the latter hypothesis will be challenging as we could detect low levels of CCL2 transcripts immediately following PBMC isolation plausibly due to concurrent cellular stimulation (data not shown). Giving credence to the argument that basal conditions are adequate for higher CCL2 expression from the rs1024611G-bearing allele, several studies showed increased serum CCL2 levels in absence of stimulation [10], [12], [17]. Further supporting such a notion, Karrer et al. showed that under basal conditions the CCL2 mRNA expression was four-fold higher in rs1024611 G/G homozygous fibroblasts when compared to A/G heterozygous fibroblasts and ∼eight-fold higher expression levels when compared to A/A homozygous fibroblasts [14]. Nevertheless, our data showing that AEI could be readily detected in the nascent CCL2 transcripts suggests that the allele-specific differences in expression are most likely determined at the transcriptional level.

### Transcriptional Basis for CCL2 AEI

Conflicting context-dependent results have been obtained with regard to transcriptional activities associated with reporter vectors containing rs1024611A and G polymorphisms. For example, testing the *CCL2* distal enhancer in reporter assays alone led to three different conclusions with regard to the role of rs1024611G polymorphism in determining the transcriptional strength, namely, increase, decrease or no change [16], [23], [30]. Nyquist et al. analyzed the effect of rs2857656C polymorphism that is linked to rs1024611G in reporter assays and found that there was an increase in basal as well as activation induced transcription [47]. However, the construct spanned ∼1.1 kb and did not contain the distal enhancer region. By contrast, Intemann et al. found that a 14 bp intron 1 deletion that occurs in the context of rs1024611G or rs1024611A allele may reduce transcriptional activity [36]. To minimize such context-dependent confounding, we generated constructs that span a 6 kb *cis*-regulatory region which allowed us to directly compare the role of the linked SNPs on transcriptional strength. To the best of our knowledge, the haplotype-specific constructs tested in this study encompass the longest contiguous human *CCL2 cis*-regulatory region examined till date. Surprisingly, our results suggested that the rs1024611G-bearing haplotype-specific construct had a lower transcriptional strength when compared to the wild-type rs1024611A-containing haplotype-specific construct in U87MG cell line. In contrast, we could not find significant differences in transcription strength between these two constructs when transfected into normal human astrocytes. While it has been widely accepted that reporter assays provide functional evidence for transcriptional mechanisms, it is increasingly being recognized that reporter assays alone cannot be used as proof of functional polymorphism in absence of other in vivo evidence such as AEI [48].

The lack of concordance between the AEI and the in vitro reporter assays could be because of several reasons. Reporter based assays do not account for the chromatin context of the regulatory region in question and cannot address the three dimensional interactions (“looping”) between distal and proximal regulatory sequences that play an important role in *CCL2* gene regulation [49], [50]. As AEI captures allele-specific differences in the context of the chromatin as well as genetic polymorphisms it provides a powerful alternative to the reporter based studies for highly polymorphic genes with complex and extended regulatory regions. Also we cannot rule out that additional polymorphic residues that are linked to the rs1024611G-bearing allele may lead to increased levels of expression. To evaluate such a possibility we assessed the role of two such polymorphisms, rs7210316 and rs9889296 which had an r^2^>0.9 with respect to rs1024611 in CEU population and were located ∼6.3 kb and ∼9.2 kb upstream of it (Fig. 4A and data not shown). While the region overlapping the rs9889296 was inactive in reporter assays, the region containing rs72110316 was marginally active (data not shown). In addition, both these regions show minimal epigenetic features associated with transcriptional activation and thus their contribution to CCL2 AEI may be negligible. We also cannot exclude the possibility of rs13900 influencing *CCL2* transcription as our reporter constructs lacked the 3′UTR but experiments are underway to address this possibility. In summary, the results from this study and previously published reports suggest that identification of a single functional SNP that could explain AEI of *CCL2* is going to be a challenging proposition because of an extended *cis*-regulatory region and extensive polymorphism.

### Can Differentially Binding Transcription Factors be Linked to CCL2 AEI?

Several transcription factors have been demonstrated to bind differentially to *CCL2* polymorphisms including IRF-1, PARP-1, STAT-1, Prep1/Pbx complexes [10], [29], [30], [51], in order to explain the increased CCL2 expression levels associated with the rs1024611G allele. While siRNA studies in conjunction with *in vitro* transcription assays suggest a role for Prep1/Pbx complexes in mediating haplotype specific activity of the *CCL2* reporter vectors [30], [31], more direct evidence for their role on endogenous *CCL2* transcription is still awaited. STAT-1 has been implicated in the CCL2 induction by IFN-γ and there are reported differences in STAT-1 binding between rs2857656G and rs2857656C alleles, with the former showing greater degree of binding [47]. However, the well characterized STAT-1 binding site that was previously reported to mediate the IFN-γ upregulation of *CCL2* is at position –212 relative to the start codon [52] and does not overlap with the predicted –362 STAT binding site reported by Nyquist et al. The increased transcriptional activity associated with rs2857656C allele cannot be directly attributed to differences in STAT-1 binding because of the following two reasons. First, STAT-1 binding to the –362G site could not be confirmed by super-shift assays. Second, the rs2857656G site shows a greater affinity to STAT-1 which is an activating transcription factor but the reporter construct with this haplotype exhibited a lower transcription activity [47]. Furthermore, while there is a perfect LD between rs1024611G and rs2857656C polymorphism, it will be difficult to argue that most of the effects of the rs1024611G bearing allele are mediated through differential binding of STAT-1α to the –362 site because this transcription factor is not known to be a major player in either TNF-α or IL-1β mediated upregulation of CCL2 expression. Thus till date there is no conclusive evidence for the role of specific transcription factor(s) in haplotype-specific differences in the transcriptional activity and generating AEI in CCL2. It could be speculated that cumulative effect of multiple transcription factors with activation or repressive potential binding to various *CCL2* cis-regulatory polymorphisms specifies haplotype-specific differences in transcriptional strength and ultimately determines allele-specific differences in expression.

### CCL2 AEI and Disease Association Studies

Studies on rs1024611G have implicated it in a myriad of disease phenotypes but often the findings across various cohorts and disease models were not consistent. While a number of factors such as cohort size, racial composition of the cohort, and the phenotype tested among others can influence such outcomes, robust molecular evidence can help in developing accurate models for disease pathogenesis and disabuse some of the conflicts that are often encountered in cohort studies [53]. Our analysis of allele-specific differences in *CCL2* gene expression in heterozygous individuals provides a powerful molecular link to the phenotypic effects that have been associated with rs1024611G allele and suggest that rs13900 SNP could serve as a reliable, potent, and functional marker for conducting future cohort-based studies.

A possible scenario where *CCL2* AEI can be helpful is in resolving contradictory data obtained with the association of rs1024611G with pulmonary tuberculosis. The rs1024611G polymorphism showed strong association with tuberculosis susceptibility in cohorts from North-East Asia, Zambia, Central America, and South America [28], [54], [55] whereas such association was not detected in studies conducted in South-East Asian, South African Colored and Indian populations [56], [57], [58]. In contrast to the above mentioned studies, Thye et al. found that rs1024611G conferred strong protection to TB in a Ghanaian cohort [59]. Feng et al. speculated that such differences may be attributed to LD between rs1024611 and an adjacent causal SNP [60]. Thus it might be helpful to study *CCL2* AEI in a population specific manner to assess whether it can explain the phenotypic differences seen in cohort-based studies thus providing a rational basis for such inconsistencies. However, other explanations for the observed race-specific differences in disease outcome are possible as polymorphisms in other gene loci have been shown to influence serum CCL2 levels [19], [20].

### Conclusion

In summary, we demonstrated here that the rs1024611 regulatory region polymorphism is associated with allelic expression imbalance of CCL2. Our study provides a molecular explanation for higher CCL2 expression levels that have been associated with the rs1024611G polymorphism and increased susceptibility of persons carrying this allele to a multitude of diseases that are thought to be mediated by the cells of monocytic lineage. Our functional studies also support the growing notion that reporter-based transcriptional studies should not serve as the sole basis to evaluate the role of *cis*-regulatory region polymorphisms on gene expression and whenever possible should be supported by in vivo expression analysis in an appropriate cell type. Functional studies such as those described in this report will help in accurately assessing the role of pathogenic alleles that have been identified by genome-wide association studies or candidate gene association studies and provide a rational explanation for such associations.

## Materials and Methods

### *CCL2* Genetic Analysis

Genotype data was downloaded from ∼25 kb that spanned the rs1024611 polymorphism from Yoruba in Ibadan, Nigeria (YRI), Japanese in Tokyo, Japan (JPT), Han Chinese in Beijing, China (CHB) and CEPH (Utah residents with ancestry from northern and western Europe) (CEU) populations (HapMap data release #28 August 2010 on Build 36). Only unrelated individuals were used for the analysis. LD maps were constructed using the JLIN (Java-based linkage disequilibrium plotter) program [61]. Exact tests were performed to identify departure from Hardy-Weinberg (HW) equilibrium. Haplotypes were obtained by using the best calculated genetic phases with the ELB algorithm, and pairwise LD was examined by calculation of the D’ and r^2^ coefficients estimated using the Arlequin software package (version 3.5.1.3; 2011) [62].

### Cell and Tissue Sources

HeLa, U87MG, and HL60 cell lines were obtained from ATCC and were maintained under recommended conditions. Primary human astrocytes were obtained from a commercial source and were cultured according to the manufacturer’s recommendation (Lonza). PBMC were isolated from healthy volunteer donors using Ficoll-Hypaque and resuspended in RPMI containing 10% FBS at 1×10^6^ per ml. PBMC were either left untreated or treated with LPS (Sigma; 1 µg/ml) for 3 h and total RNA (RNAqueous 4PCR kit; Ambion) and genomic DNA were isolated**.** Total RNA was treated with DNaseI to remove any genomic DNA contamination and was quantified using a Nanodrop spectrophotometer. The 260/280 ratios ranged between1.8–2.0. Monocytes were purified from freshly isolated PBMC using Pan Monocyte Isolation Kit (Miltenyi Biotec) according to manufacturer’s recommended protocol and their purity was>85%. Brain samples from anonymous HIV-1 infected and normal donors were obtained from National Neurological AIDS Bank, The Manhattan HIV Brain Bank, and Texas Repository for AIDS Neuropathogenesis Research that are components of the National NeuroAIDS tissue consortium (NNTC). RNA and DNA were extracted from the brain tissues by using the Trizol (Invitrogen) method according to manufacturer’s recommended protocol. Genotyping for rs1024611 polymorphism was performed as previously described [10].

### Ethics Statement

All studies on human subjects were approved by the Institutional Review Board of the University of Texas Health Science Center, San Antonio and South Texas Veterans Health Care System. All participating volunteer donors signed a written consent form which was approved by the IRB.

### cDNA Synthesis and Quantitative RT-PCR (qRT-PCR)

cDNA was synthesized (iScript cDNA synthesis kit, Biorad) using random primers according to manufacturer’s recommended protocol. Expression of CCL2 in the LPS or TNF-α stimulated cells was confirmed by qRT-PCR (Applied Biosystems assay numbers Hs00237140_m1 for CCL2 and Hs99999901_s1 for 18S). Data were normalized using the ΔΔCt method using 18S as a normalizer. The 18S rRNA levels were not significantly different between the untreated and LPS treated samples. No amplification was detected in control reactions with no added template and in reverse transcriptase negative samples.

### Capture of Nascent RNA

Nascent RNA was isolated using Click-It Nascent RNA kit (Invitrogen) to capture newly synthesized RNA according to manufacturer’s recommended protocol. Briefly, PBMC from a heterozygous individual were stimulated with LPS and incubated with ethylene uridine (EU) which is an alkyne–modified nucleoside and a uridine analog. Total RNA was isolated using RNAqueous®-4PCR Kit (Ambion). The EU-labeled RNA is biotinylated with using a “click reaction” with an azide-modifed biotin. This reaction generates a biotin-based handle on RNA molecule that is used to capture the nascent RNA transcripts on the streptavidin magnetic beads. The captured RNA was used for cDNA synthesis (Superscript Vilo kit, Invitrogen), PCR amplification, and pyrosequencing.

### Pyrosequencing

The region encompassing the rs13900 SNP was amplified by PCR in a 50 µl reaction mixture containing 2 µl cDNA, 1.5 µl 50 mM MgCl_2,_ 5 µl 10X PCR Buffer II, 1 µl 10 mM dNTP, 0.25 µl AmpliTaq DNA polymerase (Applied Biosystems), and 8 µM each of forward and reverse primers. The nucleotide sequence of the forward primer used was 5′-CCCAAGAATCTGCAGCTAACTTAT-3′ and the biotinylated reverse primer was 5′-GGCATAATGTTTCACATCAACAA-3′. The following temperature conditions were used: 94°C for 2 min followed by 35 cycles of 94°C for 10 s, 58°C for 30 s, 72°C for 1 min, and 72°C for 7 min. The nucleotide sequence of the oligonucleotide used for pyrosequencing is 5′-CTTTCCCCAGACACC-3′. Pyrosequencing was performed by a commercial firm (EpigenDx, Hopkinton, MA).

### Cloning, Transfections, and Reporter Assays

Haplotype specific constructs that contain the rs1024611A and rs1024611G polymorphisms were cloned from a heterozygous donor who exhibited AEI. For generating the haplotype-specific constructs, a 6 kb region was PCR amplified (Elongase, Invitrogen). The nucleotide sequences of the oligonucleotides used for amplification are 5′-AGCTGAGGCCCTGGTTGATTCT-3′ (forward) and 5′-GCTGGAGGCGAGAGTGCGAG-3′ (reverse). The conditions for the amplification are 94°C for 2 minutes, 35 cycles of 94°C for 10 seconds and 68°C for 6 min. The amplified fragment was cloned into pGL4.16 reporter vector (Promega) and their nucleotide sequence was verified by sequencing. Transfections into U87MG cell line, TNF-α treatment, and luciferase assays were performed as described previously [49]. Normal human astrocytes were transfected using Fugene HD (Roche) according to manufacturer’s recommended protocol. Luciferase values were normalized using the total protein content of the lysates as we found that use of the routinely used *Renilla* vector modulated the expression of the haplotype-specific reporter constructs that led to spurious results.

## Supporting Information

Figure S1 **LPS induction of CCL2 in PBMC.** Freshly isolated PBMC were either left untreated or treated with LPS for 3 h and total RNA extracted as outlined in the *Methods*. qRT-PCR was used to assess CCL2 expression and fold increase following LPS treatment *versus* the untreated samples was calculated using the ΔΔCt method. 18S rRNA was used as a normalizer. Data shown are from two independent donors and statistical significance was calculated using Student’s *t* test (*, p = 0.01, **p = 0.03).
(EPS)

Figure S2 **CCL2 AEI in purified monocytes.** Monocytes were purified from freshly isolated PBMC using Pan Monocyte Isolation Kit (Miltenyi Biotec) according to manufacturer’s recommended protocol. Total RNA was extracted from LPS treated unfractionated PBMC or purified monocytes and allelic quantification was performed on the RT-PCR products by pyrosequencing. Data shown are from two independent donors. Statistical significance was calculated using a paired t-test (*, p<0.0001).
(EPS)

Figure S3 **Transcriptional Activity of** ***CCL2*** **reporter vectors in normal human astrocytes.** Primary human astrocytes were transfected with the indicated reporter vectors and were left untreated (panel A) or treated with TNF-α (panel B). The cells were lysed after 24 h after transfection and luciferase values were measured as indicated in the *Methods*. Data is from three independent experiments each done in duplicate. Statistical significance was calculated using Student’s *t* test and was found not to be significant (p>0.3).
(EPS)
